# QUALIPAED—A retrospective quality control study evaluating pediatric long axial field-of-view low-dose FDG-PET/CT

**DOI:** 10.3389/fnume.2024.1398773

**Published:** 2024-06-13

**Authors:** Sabrina Honoré d’Este, Flemming Littrup Andersen, Christina Schulze, Eunice Saxtoft, Barbara Malene Fischer, Kim Francis Andersen

**Affiliations:** ^1^ Department of Clinical Physiology and Nuclear Medicine, Rigshospitalet, Copenhagen University Hospital, Copenhagen, Denmark; ^2^ Department of Clinical Medicine, Faculty of Health and Medical Sciences, University of Copenhagen, Copenhagen, Denmark; ^3^ King’s College London & Guy’s and St Thomas’ PET Centre, St Thomas’ Hospital, London, United Kingdom

**Keywords:** total-body PET/CT, LAFOV, pediatric imaging, low-Dose 2-[18F]FDG, Quadra

## Abstract

**Introduction:**

Pediatric patients have an increased risk of radiation-induced malignancies due to their ongoing development and long remaining life span. Thus, optimization of PET protocols is an important task in pediatric nuclear medicine. Long axial field-of-view (LAFOV) PET/CT has shown a significant increase in sensitivity, which provides an ideal opportunity for reduction of injected tracer activity in the pediatric population. In this study we aim to evaluate the clinical performance of a 2-[^18^F]FDG-tracer reduction from 3 MBq/kg to 1.5 MBq/kg on the Biograph Vision Quadra LAFOV PET/CT.

**Materials and methods:**

The first 50 pediatric patients referred for clinical whole-body PET/CT with 1.5 MBq/kg 2-[^18^F]FDG, were included. A standard pediatric protocol was applied. Five reconstructions were created with various time, filter and iteration settings. Image noise was computed as coefficient-of-variance (COV = SD/mean standardized-uptake-value) calculated from a spherical 20–50 mm (diameter) liver volume-of-interest. Sets of reconstructions were reviewed by one nuclear medicine physicians, who reported image lesions on a pre-defined list of sites. Paired comparison analysis was performed with significance at *P_B_ < 0.05* (Bonferroni corrected).

**Results:**

All reconstructions, except one, achieved a COV_mean_ (0.08–0.15) equal to or lower than current clinical acceptable values (COV_ref_ ≤ 0.15). Image noise significantly improved with increasing acquisition time, lowering iterations (i) from 6i to 4i (both with five subsets) and when applying a 2 mm Gauss filter (*P_B_ < 0.001*). Significant difference in lesion detection was seen from 150s to 300s and from 150s to 600s (*P_B_ = 0.006–0.007*). 99% of all lesions rated as malignant could be found on the 150s reconstruction, while 100% was found on the 300s, when compared to the 600s reconstruction.

**Conclusion:**

Injected activity and scan time can be reduced to 1.5 MBq/kg 2-[^18^F]FDG with 5 min acquisition time on LAFOV PET/CT, while maintaining clinical performance in the pediatric population. These results can help limit radiation exposure to patients and personnel as well as shorten total scan time, which can help increase patient comfort, lessen the need for sedation and provide individually tailored scans.

## 1 Introduction

It is theorized that due to ongoing development and long remaining life span, pediatric patients run a higher risk of radiation induced malignancies (1–9). As a result pediatric positron emission tomography (PET) protocols are constantly under investigation for ways to optimize the level of radiation exposure and reduce injected tracer activity according to the principles of As-Low-As-Reasonable-Achievable (ALARA). Current international recommendations from the European Association of Nuclear Medicine (EANM) dosage card and the North American Consensus Guidelines reports an administered activity of ≈3–5 MBq/kg 2-[^18^F]fluoro-2-deoxy-D-glucose (2-[^18^F]FDG), with a minimum of 26 MBq in pediatric patients (10, 11).

The development and following implementation of long axial field-of-view (LAFOV) PET/Computed tomography (CT), has led to a multitude of studies, which addresses the improved sensitivity as well as the subsequent possibilities. These include the feasibility of low activity and even ultra-low activity PET/CT scans (12–20). Simulated Low-Dose (LD) reconstructions generated on the uEXPLORER Total-Body (TB) PET/CT, have achieved clinically satisfactory image quality in 10 min acquisition time, with an injection of 0.37 MBq/kg 2-[^18^F]FDG in pediatric patients (17). True ultra-LD PET/CT has primarily been seen in the adult population, with clinically acceptable image quality at 0.37–0.57 MBq/kg 2-[^18^F]FDG in only 8–12 min acquisition time (14, 15, 18).

Research into true LD as well as ultra-fast PET scans with 2-[^18^F]FDG in the pediatric population remains sparse. In a case report from 2023, a group successfully performed a PET/CT on a 10 week old baby without the aid of sedation, achieving image quality capable of imaging sites of infection (12). The scan was performed on the Biograph Vision Quadra, with a total scan time of 3 min and 12 MBq 2-[^18^F]FDG, which is less than half of the minimum required dosage according to the EANM dosage card (10). A large study, performed on the uEXPLORER, included 100 mixed oncologic pediatric patients who underwent TB PET/CT with 1.85 MBq/kg 2-[^18^F]FDG tracer activity. They found that sufficient image quality and overall lesion conspicuity for clinical use could be achieved at as low as 60s acquisition time (19).

Based on previous findings (21), the clinical pediatric LAFOV PET/CT imaging protocol was updated at our facility in 2022. This lowered administered 2-[^18^F]FDG tracer activity from 3 MBq/kg to 1.5 MBq/kg. This study aimed to evaluate the clinical performance of the protocol. We sought to investigate the consequences of various reconstructive settings concerning filter application, number of iterations and acquisition time, through measurements of image noise as well as reports of lesion detection and classification.

## 2 Materials and methods

### 2.1 Study design

The study is a retrospective quality control study, performed in a tertiary hospital setting. It evaluates the clinical performance of a pediatric whole-body (WB) PET/CT protocol with an administered 2-[^18^F]FDG tracer activity of 1.5 MBq/kg. The project was approved by the Departmental Review board on January 19, 2023, app.no. 544_22. No formal consent is needed for retrospective quality assurance projects according to Danish law.

### 2.2 Study population

**Figure 1:**
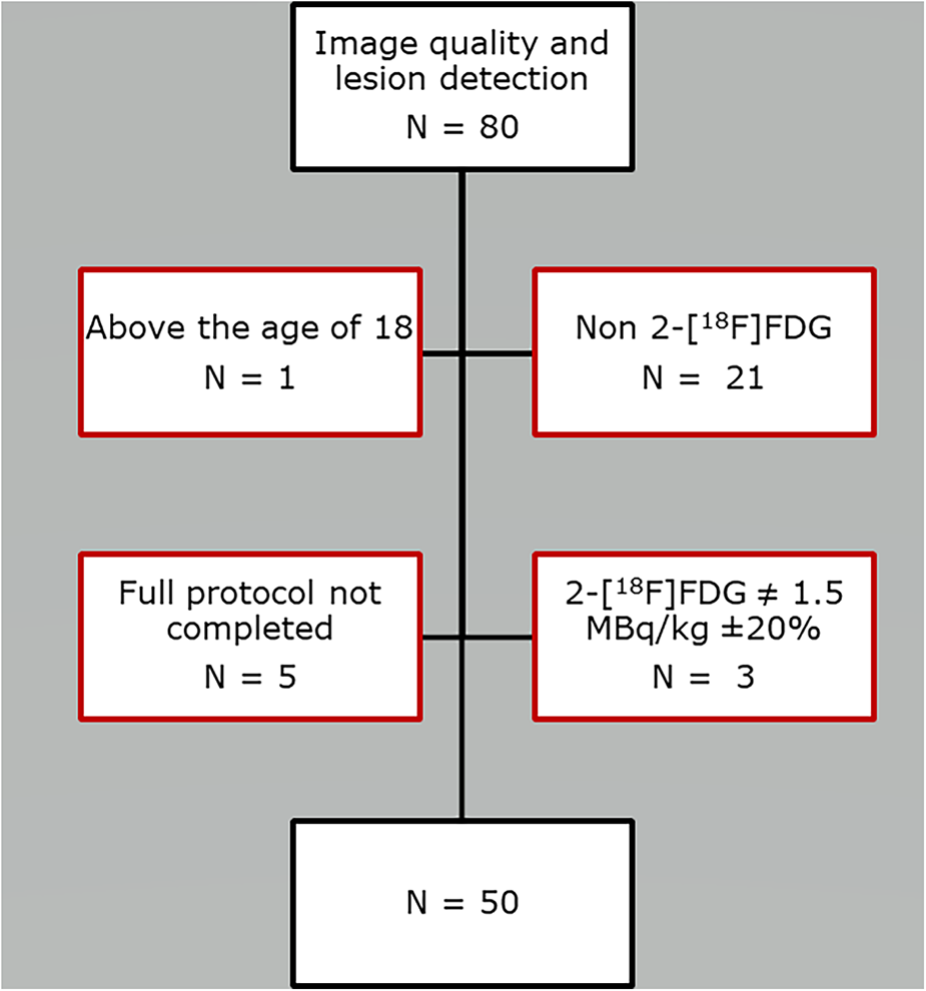
Flowchart depicting patient exclusion.

The first 50 pediatric patients (<18 years of age) referred for clinical WB PET/CT on the Biograph Vision Quadra, were included for evaluation of image quality and lesion detection (May 2022 to Jan 2023). See Table 1 for full demographics content. In- and exclusion of patients are depicted in Figure 1. A total of 80 patient scans were assessed, for inclusion of 50 scans for the image quality and lesion detection study (Figure 1).

**Table 1:** Demographics table for image quality and lesion detection.

Characteristic	Value (*n* = 50)
Age (y = years)	
<2y (*r* = 10 mm)	1
2–6y (*r* = 15 mm)	6
6–12y (*r* = 20 mm)	14
>12y (*r* = 50 mm)	29
Sex	
Male	27
Female	23
BMI (kg/m^2^)	19.1 ± 3.63
Radiation	
Injected activity (MBq)	
Injected activity per weight (MBq/kg)	1.53 ± 0.08
Effective dosage (mSv)	1.10–3.22
Sedation	
General anesthesia	6
Pathology	
Lymphoma	11
Sarcoma	9
Other malignancies	18
Infection	6
Autoimmune	6
Field of view (FOV)	
1 FOV	38
2 FOV	12

### 2.3 Imaging

All patients were confirmed to have a plasma-creatinine within normal limits, preceding the PET/CT with intravenous contrast. Patients were required to have fasted for minimum of 4 h prior to scanning. Following 60 min rest post-injection of 1.5 MBq/kg 2-[^18^F]FDG ±20%, a low-dose CT scan was performed. Sedation was applied when necessary for satisfactory cooperation (Table 1). Patients were predominantly positioned in a vacuum fix pillow supplemented with a light fixation across the body using a hook-and-loop belt with arms free. Next of kin was allowed to be present in the room if deemed necessary for cooperation. PET data was processed acquired using a standard clinical protocol of: 4–6iterations (i) with 5subsets, 1.65 × 1.65 mm voxels, Point-Spread-Function, Time-of-Flight modeling with maximum ring difference of 85, 106 cm fixed scan, one or two bed positions, and 600s acquisition time. A post-processing 2 mm Gauss filter was applied. The following five reconstructions were recreated from the total list mode PET data stream from scan start until the given time point and included in the systematic evaluation; (A) Gauss Filter, 4i, 150s (F150s); (B) Gauss Filter, 4i, 300s (F300s); (C) Gauss Filter, 4i, 600s (F600s); (D) No Filter, 4i, 300s (N4i300s) and (E) No Filter, 6i, 300s (N6i300s).

### 2.4 Image quality

A 20–50 mm (age dependent diameter, see Table 1) spherical VOI (right liver lobe avoiding major arteries or pathologies) was placed on the CT, by the author. The VOI was copied on to the five reconstructions (A-E), and standardized-uptake-value (SUV) mean and max as well as SD were extracted. Image quality was computed as coefficient-of-variance (COV = SD/SUV_mean_) with 0.15 considered equal to current standard of care in a clinical 2-[^18^F]FDG PET/CT scan (22–25).

### 2.5 Lesion detection and classification

All scans were reviewed by one nuclear medicine physician, with more than ten years of experience in pediatric PET-imaging. All scans were blinded except for age, sex and a shortened referral text, for instance: “Primary staging: Hodgkin's Lymphoma”. A complete list of the presented patient data can be found in Supplementary 1.1. Each set of scans included five image reconstructions (A-E) as well as the relevant CT, always beginning with the shortest acquisition period (150s), ending with the longest (600s). The total number of image findings (lymph nodes and organ lesions) had to be reported on a pre-defined list of sites (Supplementary 1.2) for evaluation of lesion detection. In addition, the image findings had to be characterized as “benign”, “equivocal” or “malignant” for evaluation of diagnostic certainty of lesion classification. Any lesions, defined as any type of image lesion finding, which could be confidently identified, were included. The number of findings was noted up to a maximum of five at each site. The full count F600s reconstruction was used as reference for lesion detectability.

### 2.6 Statistical analysis

All statistical analysis and data presentation were performed using Microsoft Excel 2013 and IBM SPSS Statistics 28. Statistical significance was achieved with a *P* value <0.05 and Bonferroni correction was applied for all possible pairings when multiple comparisons were performed (PB). Data was divided into two groupings; time dependent reconstructions (F150s, F300s and F600s) and filter/iteration dependent reconstructions (F300s, NF4i300s and NF6i300s) for comparison. Analysis of variation or Friedman's test was performed for all data points and further examined by paired comparison analysis (*t*-test and Wilcoxon signed rank test), when significant. An overview of statistical results can be seen in Supplementary 2.1. A simple percentage was also calculated during the evaluation of lesion classification.

## 3 Results

### 3.1 Demographics

Some children received follow-up scans during the inclusion period, which resulted in a total of 16 individual female patients (23 scans) and 24 male patients (27 scans). The children included had a mean age of 12 ± 5 years (Table 1).

### 3.2 Image quality

**Figure 2:**
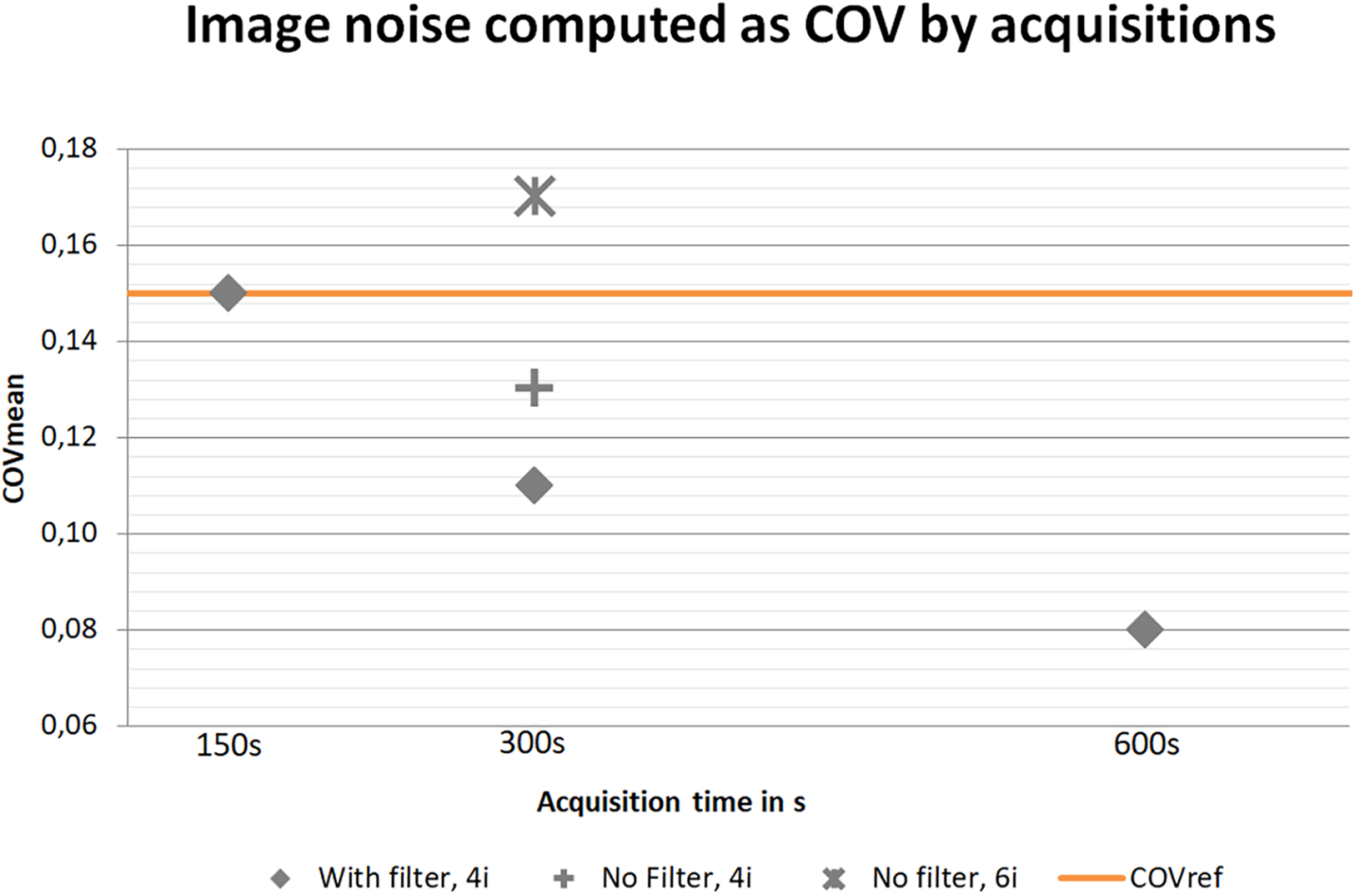
Image noise computed as COV_mean_ by acquisition time. Orange: reference maximum level of noise accepted according to current standard of care in a clinical PET/CT scan.

Due to a surgical implant artefact, a 30 mm VOI was applied in one patient despite being 16 years of age. All reconstructions, except NF6i300s, achieved a COVmean equal to or lower (COV_mean_ = 0.08–0.15) than current acceptable clinical values at our institution (COV_ref_ ≤ 0.15) with the F600s providing the least image noise, COV_mean_ = 0.08 (See Figure 2 for mean values of image noise computed as COV). The repeated measures ANOVA showed significant difference between reconstructions with a *P* < 0.001. Paired samples t-test for both the time dependent group: F150s, F300s and F600s, and the filter/iteration dependent group: F300s, NF4i300s and NF6i300s, showed significant difference between all three pairs in each group (*P_B_* < 0.001).

### 3.3 Lesion detection

**Figure 3:**
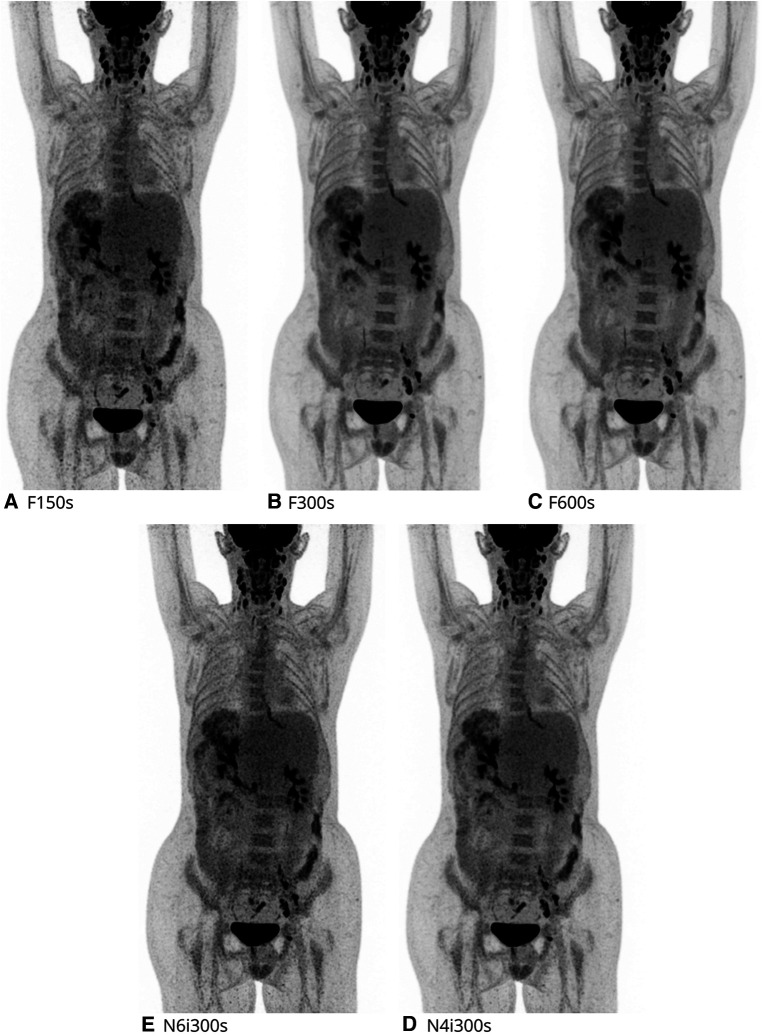
2-[18F]FDG maximal intensity projection (MIP) of each reconstruction evaluated. Here a 16y male, referred for recurrence of post-transplant-lymphoproliferative-disease.

All statistical tables can be found in Supplementary 2. The total lesion count (all 43 anatomical locations) was significantly different, across all five reconstructions (*P* < 0.001). The anatomical locations were divided into those of organs (anatomical location no. 1–30) and lymph nodes (anatomical location no. 31–43). For the organ lesion count, no significant difference in lesion detection was seen (*P* = 0.837). For the lymph nodes, a significant difference in lesion detection was seen (*P* < 0.001). Thus, further analysis of the time dependent and filter/iteration dependent reconstructions was performed for the total lesion count and the lymph nodes. See Figure 3 for exemplar single set of scans across reconstructions.

#### 3.3.1 Acquisition time

**Figure 4:**
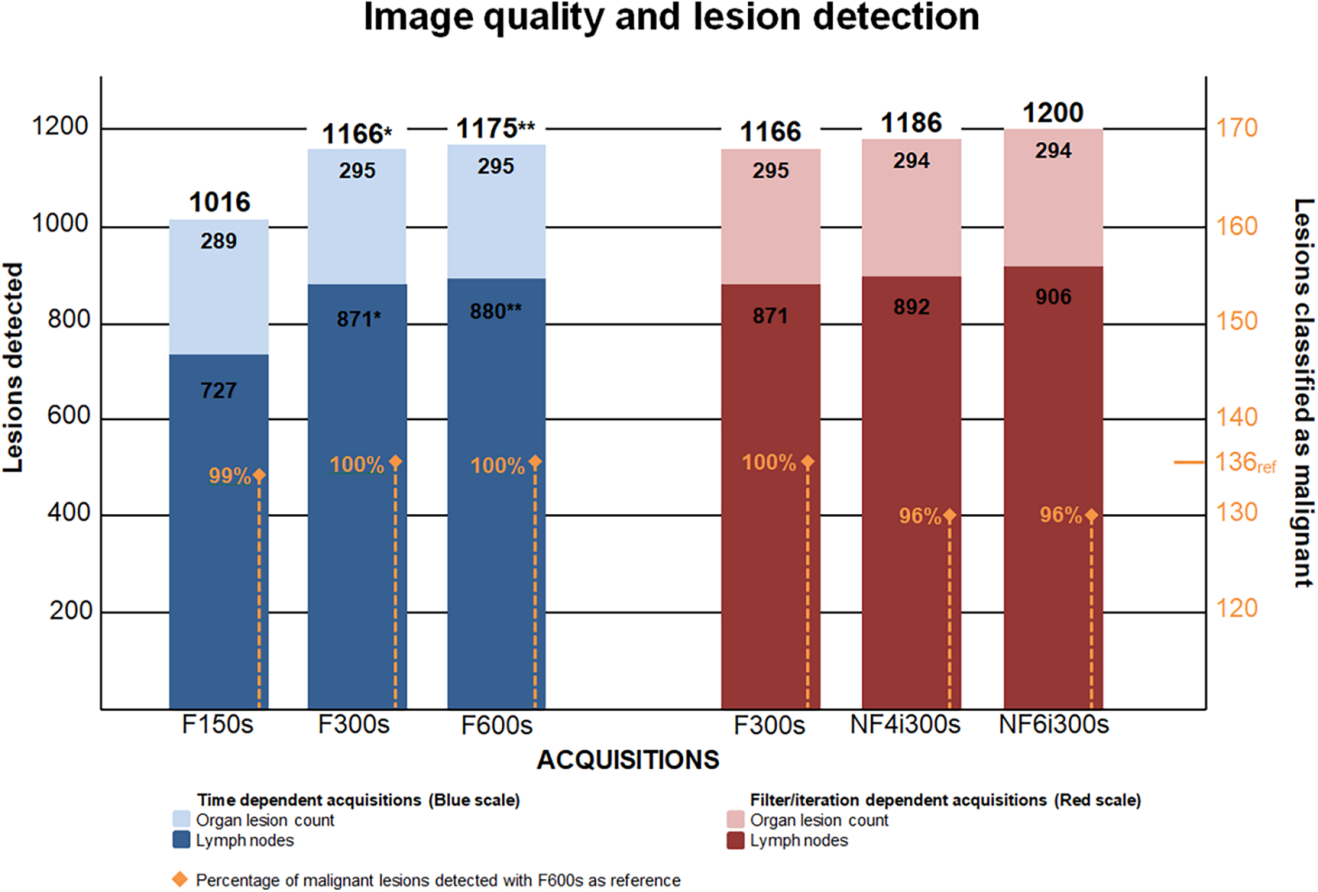
Depicts the number of lesions detected and compared in the time dependent and filter and iteration dependent reconstructions. *: Significant compared to the previous reconstruction. **: Significant compared to the reconstruction twice removed.

Comparing images across acquisition times (F150s, F300s and F600s), we found a significant difference in the total lesion count (*P* < 0.001) as well as when comparing the lymph nodes (*P* < 0.001). Pairwise comparison demonstrated a significant difference in total lesion count from F150s to F300s (*P_B_* = 0.007) and from F150s to F600s (*P_B_* = 0.006), while no significant difference was observed from F300s to F600s (*P_B_* = 0.512). Concurring results was found when we compared lymph nodes, with a significant difference from F150s to F300s and from F150s to F600s (*P_B_* = 0.019), but not from F300s to F600s (*P_B_* = 0.512) (Figure 4).

#### 3.3.2 Filter and iterations

There was no significant difference in lesion detection between the filter and iteration dependent reconstructions (F300s, NF4i300s and NF6i300s) (*P* = 0.058). However, a difference was seen when comparing the number of lymph nodes detected (*P* = 0.045), which did not reach significance in any of the pairwise comparisons (Figure 4).

### 3.4 Lesion classification

**Figure 5:**
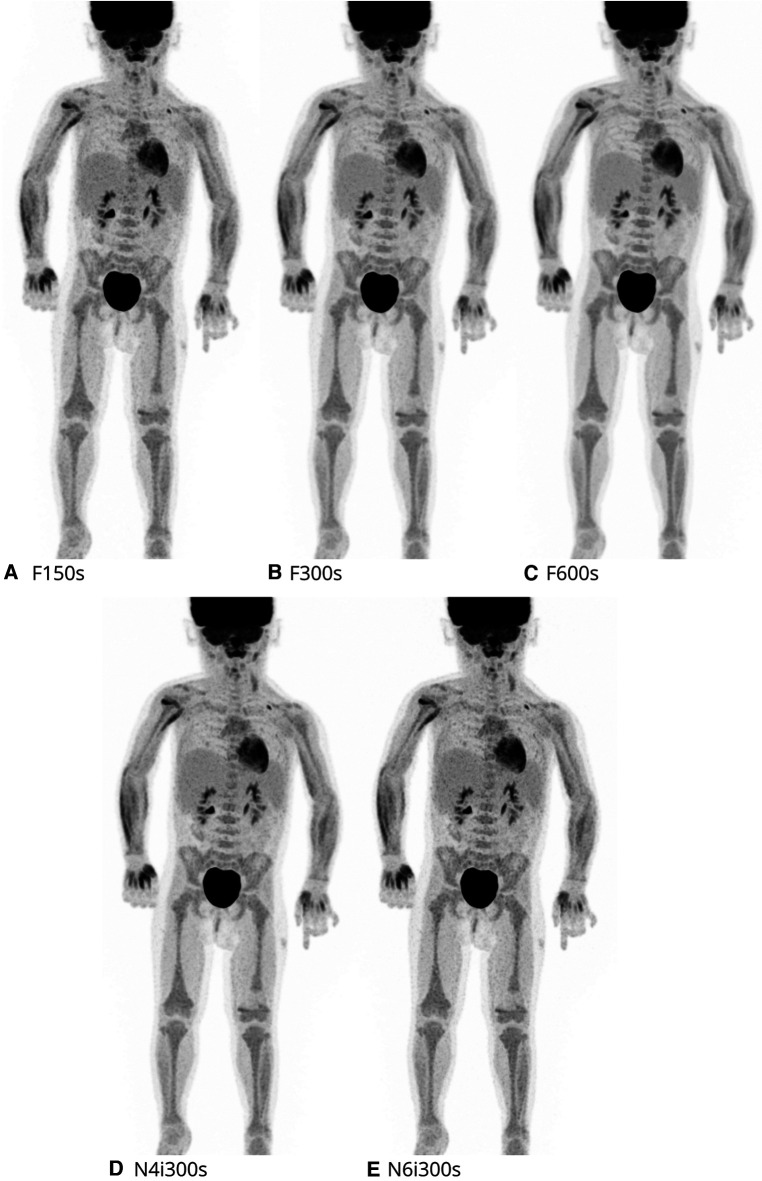
2-[18F]FDG maximal intensity projection (MIP) of each reconstruction evaluated. Here a 2y female, referred for evaluation of treatment of alveolar rhabdomyosarcoma.

No lesions changed classification across any of the five reconstructions and no significant difference in malignant lesion findings was observed (*P* = 0.866). All lesions rated as malignant were pairwise compared using F600s as the reference. 100% (136/136) of these could be found on the F300s, 99% (134/136) on the F150s while only 96% (130/136) of malignant findings were detected when the Gauss filter was removed and the total number of iterations increased from four to six (both with five subsets). See Figure 5 for exemplar single set of scans across reconstructions.

## 4 Discussion

After the implementation of a pediatric WB PET/CT protocol of 1.5 MBq/kg 2-[^18^F]FDG tracer activity on the Biograph Vision Quadra LAFOV PET/CT, we evaluated the clinical performance of various reconstructive settings. We found no difference in clinical performance through changes to filter and iteration settings or through a reduction in acquisition time from 10 min to 5 min, although image quality did improve within the same time frame. This suggested that no proportional adjustment in scan time was needed to accommodate for the lowered injected activity on LAFOV PET/CT and is in keeping with previously published studies (12–14, 16–19, 26–28).

### 4.1 Structural imaging

Diagnostic nuclear imaging in children will always pose numerous challenges, typically centered on the potential carcinogenic effects from the radiation exposure of the injected tracer. With the increased sensitivity, made possible with LAFOV PET, the injected activity can be halved without compromising the diagnostic performance. In an average five-year old child undergoing a 2-[^18^F]FDG PET scan, this would mean a reduction in applied effective dose of radiation to almost half of the average yearly background radiation and a fraction of a standard diagnostic CT scan (29–33). With the PET radiation exposure per scan reduced to near inconsequential levels, patients as well as lab technicians and other personnel can remain at a lower risk of radiation ramifications. While this follows the ALARA principle, the overall effect might be questionable as long as clinical PET imaging remains dependent on the CT scan and its relatively high additional radiation exposure. This could be prevented by replacing the PET/CT with PET/MRI, which have also shown promising results in regards to 2-[^18^F]FDG tracer activity reduction in pediatric oncology (34). Further, complete elimination of the CT through application of artificial intelligence might also prove to be an interesting solution as technical advances continues to grow (35).

### 4.2 Lower and faster with LAFOV PET/CT

Previous studies using a true reduction in injected activity, as opposed to a modelled reduction, have showed acceptable image quality in adult lung-cancer and melanoma patients at 1.85–2.00 MBq/kg 2-[^18^F]FDG, while maintaining a total scan time of ≈5 min (13, 16). Further, satisfactory image quality in adult colorectal cancer patients have been achieved at just 0.37 MBq/kg in 8 min acquisition time (14). While the difference in age and metabolism in adult vs. pediatric populations has an impact on dosimetry and image conspicuity (36–40), our results concurred with theirs, which further confirms the uprising possibilities of LAFOV PET/CT.

In 2022 the first pediatric study with a reduced activity of injected 2-[^18^F]FDG dosage was published. 100 patients (age 1y–13y) were injected with 1.85 MBq/kg for an estimated effective dose of 1.76–2.57 mSv, and scanned on the uEXPLORER TB PET/CT. It was proven that half dose 2-[^18^F]FDG could provide clinically usable images in only 60s (19). Compared to this our results appear contradictory. While we did find clinically satisfactory image quality at 150s, we found that lesion detection could be improved by increasing the acquisition time to 300s, ultimately bettering the diagnostic performance. This may be explained by the large heterogeneity of our population due to broader age span (4m–17y) and effective dose (1.10–3.22 mSv) as well as inclusion of non-malignant referrals. However, the higher sensitivity of the uEXPLORER due to the increased AFOV of 194 cm compared to the Biograph Vision Quadra of 106 cm, may also contribute to the difference in results (41, 42).

Another great challenge of pediatric diagnostic imaging is lack of compliance. This is typically one of the most time-consuming challenges and has the potential of putting a significant delay on the daily schedule. At half of recommended 2-[^18^F]FDG tracer activity, we can achieve the same diagnostic performance in 10 min as we can in 5 min. Further, 99% of malignant findings were visible after just 2.5 min acquisition time, which is promising for a potential further reduction in PET acquisition time. Ultimately, this can help limit total scan time to less than 10 min included CT and aid to reduce anxiety, reduce the need for sedation as well as improve patient comfort in the pediatric population. Case reports have already been presented, which shows LAFOV PET/CT have been successfully performed on a 10 week infant as well as at 1.5years without the need of sedation (12, 27).

### 4.3 Strengths and limitations

The continued principle of ALARA and possibilities of LAFOV PET/CT results in administration of extremely small amounts of tracer activity. This of course presents certain difficulties in regards to the residual tracer left in the syringe, which becomes proportionally greater. For this study, with an administered activity of 11.5 MBq at the lowest, we thus had to accept a variation in injected 2-[^18^F]FDG tracer of 1.5 MBq/kg ±17% (1.36–1.75 MBq/kg). This could prove to become a liability in future paradigms which may need specific levels of activity. The principal limitation of the study centers on the use of a single observer, which increases risk of bias. This was sought to be overcome by the physicians' extended experience within 2-[^18^F]FDG PET, especially with analysis of pediatric PET images. Further, reconstructions were analyzed in consecutive order always starting with the F150s reconstruction, to minimize potential copy of lesions from one scan to another. We acknowledge the risk of type-II error is increased due to our application of the conservative Bonferroni correction. The correction had an impact on significance in the case of lesion detection from F300s to F600s. However, with an initial borderline insignificance this was not deemed relevant for the conclusions of the study (Supplementary 2). Image quality results remained indifferent. At last we believe our results remain at value and are relevant for a large pediatric population, as provided by the wide inclusion criterion.

### 4.4 How low can we go?

A new software update has recently become available, which utilizes the full maximum ring difference of 322 as compared to the currently available 85 rings and allows for continuous bed motion. The implementation of such advances will further increase the sensitivity and presumably result in even faster scan times and lower tracer activity. However, with increasingly lower activity, further research in terms of how to ensure uniform bio distribution of such extreme low quantities will also be needed. So the question remains; how low can we actually go?

## 5 Conclusion

Injected 2-[18F]FDG tracer activity can be reduced to 1.5 MBq/kg with 5 min acquisition time per FOV on LAFOV PET/CT, while maintaining clinical performance in the pediatric population. Subjective ratings of lesion classification indicate that further reduction at 2.5 min may be plausible; however, additional research will have to confirm this. These results can help limit radiation exposure in patients/personnel and shorten total scan time. Ultimately, achieving an increase in patient comfort, a reduced need for sedation and facilitating the concept of individually tailored examinations.

## Data Availability

The raw data supporting the conclusions of this article will be made available by the authors, without undue reservation.
