# Supplementary material for: QUALIPAED—A retrospective quality control study evaluating pediatric long axial field-of-view low-dose FDG-PET/CT
Source: Front Nucl Med. 2024 Jun 13;4:1398773. doi: 10.3389/fnume.2024.1398773 (PMC11440848; doi:10.3389/fnume.2024.1398773)
Supplement: Supplementary file 1 [file Datasheet1.pdf]

Supplementary Material

1 Supplementary Data

1.1 Age & referral

16y	*Recurrence, Post-Transplant Lymphoproliferative Disorder (PTLD)*
17y	*Treatment control, bilateral germ cell tumors in the ovaries + multiple vascular thrombi*
5y	*Treatment control, adrenocortical carcinoma with liver recurrence*
10y	*Recurrence, hepatoblastoma + residual disease in scapula*
13y	*Primary diagnosis, vertebral tumor with neuroforaminal involvement*
15y	*Recurrence, anaplastic large cell lymphoma*
15y	*Treatment control, mediastinal germ cell tumor + thrombosis of subclavian vein*
10y	*Primary diagnosis, occult tumor in the lung*
14y	*Primary diagnosis, occult malignancy*
15y	*Treatment control, nasopharyngeal cancer*
15y	*Treatment control, disseminated and refractory ostegenic sarcoma*
10y	*Primary diagnosis, osteomyelitis in the jaw + tuberculosis*
2y	*Primary diagnosis, occult cancer in the small pelvis*
10y	*Treatment control, lymphoma*
13y	*Primary diagnosis, occult tumorin thoracic vertebra (Th1) seen on MR*
16y	*Treatment control, melanocytic tumor*
10y	*Recurrence, hepatoblastoma*
13y	*Recurrence, sarcoidosis*
10y	*Treatment control, Burkitt's lymphoma*
10y	*Primary diagnosis, Hodgkin's lymphoma + nodular sclerosis*
11y	*Treatment control, sarcoma*
17y	*Recurrence /treatment control, medulloblastoma*
8y	*Primary diagnosis, chronic lymphadenitis + intermittent fever*
16y	*Treatment control, alveolar rhabdomyosarcoma*
8y	*Primary diagnosis, abscess/endocarditis/salmonella*
14y	*Treatment control, Hodgkin's lymphoma*
7y	*Recurrence, infection with salmonella*
16y	*Primary diagnosis, lung abscess*
17y	*Primary diagnosis, residual fever*
16y	*Treatment control, Hodgkin's lymphoma*
14y	*Recurrence, lymphadenopathy + Griscelli syndrome + hemophagocytosis*
16y	*Primary diagnosis, infection of spinal implant*
17y	*Staging, lymphoma*
15y	*Treatment control, lymphoma*
15y	*Treatment control, osteogenic sarcoma*
2y	*Treatment control, alveolar rhabdomyosarcoma*
14y	*Primary diagnosis, PTLD + Epstein-Barr virus*
2y	*Treatment control, disseminated alveolar rhabdomyosarcoma*
16y	*Primary diagnosis, sarcoma*
5y	*Treatment control, adrenocortical carcinoma with liver recurrence*
6y	*Treatment control, Langerhans cell histiocytosis*
16y	*Recurrence, alveolar rhabdomyosarcoma left arm*
16y	*Treatment control, Hodgkin's lymphoma*
0.25y	*Primary diagnosis, sarcoma*
17y	*Treatment control, Hodgkin's lymphoma*
10y	*Treatment control, hepatoblastoma*
4y	*Primary diagnosis, osteomyelitis*

13y *Primary diagnosis, endocarditis with extra-cardial foci*
16y *Staging, multifocal infiltrations suspect of malignancy on MR*
10y *Treatment control, Hodgkin's lymphoma*

1.2 Total list of anatomical sites

Organs

1 Brain
2 Oro/naso/hypopharynx/larynx
3 Thyroid gland
4 Lung, right
5 Lung, left
6 Pleura, right
7 Pleura, left
8 Breast, right
9 Breast, left
10 Esophagus
11 Liver
12 Pancreas
13 Adrenal gland, right
14 Adrenal gland, left
15 Kidney, right
16 Kidney, left
17 Spleen
18 Stomach
19 Bowel
20 Peritoneal
21 Ovary, right
22 Ovary, left
23 Uterus
24 Cervix
25 Vagina/vulva
26 Prostate
27 Penis/scrotum
28 Bones
29 Cutis/skin
30 Other

Lymphnodes

31 Head/neck, right
32 Head/neck, left
33 Axilla/subpect, right
34 Axilla/subpect, left
35 Mediastinum/hilar/retrocrural
36 Int mam, precardiac
37 Retroperitoneal
38 Abdominal
39 Iliac, right
40 Iliac, left
41 Groin, right
42 Groin, left
43 Lnn, other

2 Supplementary Tables

2.1 Summary of statistical results from the evaluation of lesion detection.

SUPPL. TABLE 2.1 Summary of statistical results from the evaluation of lesion detection.

Acquisition grouping	Friedman's test	Corresponding pairs	Wilcoxon signed rank test
**Total Findings**	***Significant***		
Time dependent acquisitions	*Significant*	F150s – F300s	*Significant*
		F150s – F600s	*Significant*
		F300 – F600s	*Not significant*
Filter/iteration dependent acquisitions	*Not significant*	-	-
**Organ Findings**	***Not significant***		
Time dependent acquisitions	*NA*	-	-
Filter/iteration dependent acquisitions	*NA*	-	-
**Lymph node Findings**	***Significant***		
Time dependent acquisitions	*Significant*	F150s – F300s	*Significant*
		F150s – F600s	*Significant*
		F150s – NF4i300s	*NS*
		F150s – NF6i300s	*NS*
		F300s – F600s	*Not significant*
Filter/iteration dependent acquisitions	*Significant*	F300s – NF4i300s	*Not significant*
		F300s – NF6i300s	*Not significant*
		F600s – NF4i300s	*NS*
		F600s – NF6i300s	*NS*
		NF4i300s – NF6i300s	*Not significant*

Suppl. table 2.1: NA: Not applicable. NS: Not supplied. These pairs were included as possible pairs in the Bonferroni correction, but have no relevant outcome and are therefore not included.

2.2 Significance of Friedman's test

SUPPL. TABLE 2.2 Significance of Friedman's test

P-value of	All five reconstructions	Time dependent *F150s, F300s, F600s*	Filter/iteration dependent *F300s, NF4i300s, NF6i300s*
Total image findings	*<0.001**	*<0.001**	*0.058*
Organ findings	*0.837*	NA	NA
Lymph node findings	*<0.001**	*<0.001**	*0.045**

*Suppl. table 2.2: All P-values of the performed Friedman's test. Significance marked with *.Friedman's test was performed for the time and filter/iteration dependent acquisitions when the initial for all five reconstructions was significant. Pairwise comparison was indicated when significant values were found in the time and filter/iteration dependent groupings. NA: Not applicable*

2.3 Time dependent Wilcoxon rank test significance.

SUPPL. TABLE 2.3 Time dependent Wilcoxon rank test significance.

	Compared time dependent acquisitions		
	F150s → F300s	F300s → F600s	F150s → F600s
**Total lesions**			
Increase in lesions	+150*	+*9*	+*159**
P	*<0.001*	*0.039*	*<0.001*
P_B	*0.007*	*0.512*	*0.006*
**Organ lesions**			
Increase in lesions	+6	0	+6
P	NA	NA	NA
P_B	-	-	-
**Lymph node lesions**			
Increase in lesions	+144*	+9	+153*
P	*0.001*	*0.039*	*0.001*
P_B	*0.019*	*0.512*	*0.019*

Suppl. Table 2.3: All P-values for the paired comparison of lesion detection between the time dependent acquisitions. Bonferroni correction by a factor of 13 P-value (P_B). NA: Not applicable

2.4 Filter/iteration dependent Wilcoxon rank test significance

SUPPL. TABLE 2.4 Filter/iteration dependent Wilcoxon rank test significance

	Compared filter/iteration dependent acquisitions		
	F300s → NF4i300s	NF4i300s → NF6i300s	F300s → NF6i300s
**Total lesions**			
Increase in lesions	+20	+*14*	+*34*
P	NA	NA	NA
P_B	-	-	-
**Organ lesions**			
Increase in lesions	-1	0	-1
P	NA	NA	NA
P_B	-	-	-
**Lymph node lesions**			
Increase in lesions	+21	+14	+35
P	*0.181*	*0.017*	*0.062*
P_B	-	*0.218*	-

Suppl. Table 2.4: All P-values for the paired comparison of lesion detection between the filter and iteration dependent acquisitions. Bonferroni correction by a factor of 13 (P_B). NA: Not applicable
